# Need for Antibiotics in Cases of Acute Uncomplicated Diverticulitis: A Meta-Analysis of Conservative Versus Antibiotic Treatment Approaches

**DOI:** 10.7759/cureus.86951

**Published:** 2025-06-29

**Authors:** Gaurav Maheshwari, Sadaf Khalid, Abil Ansari, Ahmed Elmoraly, Ayesha Javaid, Muhammad Rehan Mumtaz, Zulfiqar Ali

**Affiliations:** 1 General and Colorectal Surgery, Medway Maritime NHS Foundation Trust, Kent, GBR; 2 Medicine, Medway Maritime Hospital NHS Trust, Kent, GBR; 3 General and Colorectal Surgery, Medway Maritime Hospital NHS Trust, Kent, GBR; 4 Community Medicine, Al-Nafees Medical College, Islamabad, PAK; 5 Medicine, Tehsil Headquarter (THQ) Hospital, Kharian, PAK; 6 General Surgery, Rashid Latif Medical College, Lahore, PAK

**Keywords:** antibiotics, conservative management, diverticulitis, meta-analysis, randomized controlled trial

## Abstract

Acute uncomplicated diverticulitis (AUD) is often treated with antibiotics, though recent evidence questions their necessity in clinically stable patients. Concerns over antibiotic resistance have prompted consideration of conservative, non-antibiotic management, but clinical equivalence remains debated. This study aimed to compare antibiotic versus non-antibiotic management for CT (computed tomography)-confirmed AUD in adults, focusing on outcomes like mortality, complications, recurrence, surgery, and length of hospital stay (LOS). A systematic search (2016-2025) was carried out across five databases, identifying randomized controlled trials (RCTs) comparing antibiotic to conservative treatment in patients with Hinchey 1a/1b or Modified Neff grade 0 diverticulitis. Two reviewers independently extracted data and assessed bias using the Preferred Reporting Items for Systematic Reviews and Meta-Analyses (PRISMA) guidelines. Primary outcomes were analyzed using a DerSimonian-Laird random-effects model. Seven RCTs (n = 8,035) met inclusion criteria. No significant differences were found in mortality (odds ratio (OR) 1.06; 95% CI: 0.71-1.58), complications (OR 0.67; 95% CI: 0.27-1.67), emergency surgery (OR 0.60; 95% CI: 0.23-1.54), or recurrence (OR 0.96; 95% CI: 0.65-1.44). LOS was marginally shorter in the conservative group. Heterogeneity for mortality and LOS was moderate (I² = 43.2%). Most studies defined clinical stability based on the absence of systemic signs and CT-confirmed localized inflammation; some variations in diagnostic criteria were noted. Conservative treatment without antibiotics in select AUD patients yields comparable outcomes to antibiotic use. These findings support guideline recommendations favoring selective antibiotic use, though heterogeneity in population definitions and short follow-up in some studies should be noted.

## Introduction and background

Diverticular disease of the colon, often referred to as symptomatic diverticulosis, is a common gastrointestinal condition affecting both men and women. It ranks among the top five gastrointestinal diseases in terms of healthcare expenditures in Western countries [1-3]. Clinical presentations of diverticular disease include diverticular bleeding, acute or chronic diverticulitis, segmental colitis associated with diverticulosis, and symptomatic uncomplicated diverticulosis, typically characterized by abdominal pain and altered bowel habits.

In the United States alone, approximately 2.5 million cases of diverticular disease result in nearly 300,000 hospitalizations annually [4]. While the majority of individuals with diverticulosis remain asymptomatic, an estimated 10% to 25% will develop symptomatic disease, and about 15% to 20% of these patients are diagnosed with acute diverticulitis [5-9]. Acute diverticulitis is further categorized into complicated and uncomplicated forms, with the latter, acute uncomplicated diverticulitis (AUD), comprising the majority of cases. AUD is defined by the absence of complications such as perforation, abscess, fistula, or obstruction.

Although acute diverticulitis has historically been viewed as a potentially recurrent and complicated illness, more recent evidence suggests a milder clinical course, especially in sigmoid disease [10,11]. Recurrence rates are estimated at 13% to 19%, with fewer than 5% of patients developing severe complications [5,11]. Moreover, hospitalizations, often driven by intravenous antibiotic administration, constitute the bulk of healthcare costs, with bed occupancy alone accounting for 65% to 70% of expenses [1,5].

Traditionally, AUD management centered on routine antibiotic therapy, usually initiated in-hospital. However, current international guidelines, including those from the American Gastroenterological Association, suggest that antibiotics may be reserved for selected cases, reflecting growing concern over antimicrobial resistance and healthcare overutilization [12].

Recent randomized controlled trials (RCTs) have challenged the necessity of antibiotics in stable AUD patients, proposing that conservative treatment without antibiotics may yield similar outcomes [13,14]. However, definitions of clinical stability and diagnostic criteria vary across studies, and there is a lack of large-scale meta-analyses that consolidate findings from newer trials.

This study aims to fill that gap by conducting a comprehensive meta-analysis to compare outcomes, including recurrence, complications, and mortality, between antibiotic and conservative treatment strategies in CT-confirmed AUD. This evidence could help refine future guidelines and reduce unnecessary antibiotic exposure.

## Review

Materials and methods

Search Strategy

This meta-analysis followed the Preferred Reporting Items for Systematic Reviews and Meta-Analyses (PRISMA) guidelines [15]. A comprehensive search was conducted in PubMed, Cochrane Central Register of Controlled Trials (CENTRAL), Scopus, ProQuest, and Google Scholar to identify RCTs evaluating the necessity of antibiotics in AUD. English-language studies published from January 2016 to March 2025 were included. Medical Subject Headings (MeSH) and keywords such as “Diverticulitis,” “Antibiotics,” and “Randomized Controlled Trials” were used. Retrieved citations were imported into EndNote X9 (Clarivate, Philadelphia, PA) for screening and duplicate removal.

Study Selection

Two independent reviewers screened titles and abstracts, followed by full-text assessments for inclusion. Disagreements were resolved through discussion or consultation with a third reviewer. Inclusion criteria were (1) adult patients with CT-confirmed, early, uncomplicated diverticulitis (Hinchey 1a/1b or Modified Neff grade 0); (2) RCT design comparing antibiotic therapy with conservative (non-antibiotic) management; and (3) English language or English translation available. Studies were excluded if they (1) involved pediatric populations, (2) focused on complicated diverticulitis (Modified Hinchey II or higher), or (3) were non-RCTs such as observational studies, reviews, or case reports.

Challenges during study selection included heterogeneity in terminology and overlapping patient groups across trials. Some studies lacked sufficient detail in abstracts, requiring full-text assessment, which may affect replicability for future researchers.

Data Extraction

Data extraction was independently performed by two authors using a standardized, pilot-tested form to ensure consistency and accuracy. Extracted data included publication details (authors, year), study design, patient demographics, diagnostic imaging method (e.g., CT criteria), classification system used (Hinchey/Neff), treatment arms (antibiotic regimen, duration, and route vs. conservative measures), and outcome reporting methods.

Primary outcomes included all-cause mortality, complication rates, emergency surgery, recurrence, and length of hospital stay (LOS). Complications were defined as adverse clinical events such as abscess formation, bowel perforation, gastrointestinal bleeding, obstruction, or progression to complicated diverticulitis. However, definitions for individual complications (e.g., “perforation” or “treatment failure”) varied slightly between studies, which may have influenced outcome interpretation.

Statistical Analysis and Risk-of-Bias Assessment

Statistical analysis was conducted using R Studio, version 2022.02.0-443 (Posit, Boston, MA), and the meta package. Dichotomous outcomes were pooled using odds ratios (ORs), and continuous outcomes (e.g., LOS) using mean differences (MDs), both analyzed under a DerSimonian-Laird random-effects model. Statistical significance was set at p < 0.05. Sensitivity analysis was performed by excluding the Korean trial by Kim et al. [16], which focused on right-sided diverticulitis, to assess its influence on heterogeneity and outcome direction.

Risk-of-bias assessment was carried out independently by two reviewers using the Cochrane Risk-of-Bias 2 (RoB 2) tool. Each study was evaluated for bias in randomization, allocation concealment, blinding, incomplete outcome data, selective reporting, and other sources. Due to the limited number of studies (n = 7), a formal subgroup comparison based on risk-of-bias ratings was not conducted. Overall, five studies were deemed low risk, while two had some concerns. This modest risk variation was noted but did not substantially affect our confidence in the pooled estimates.

Results

Summary of Included Studies

An extensive search of electronic databases yielded 489 records relevant to the topic. Before the screening phase, 35 duplicate records were removed. Additionally, 14 records were marked as ineligible due to language limitations, incomplete citations, or irrelevant publication types, and 96 records were excluded for other reasons, including preliminary studies, conference abstracts, and non-human research. This resulted in 344 records being screened by title and abstract.

Following the initial screening, 179 records were excluded for not meeting the inclusion criteria. The remaining 165 records were selected for full-text retrieval. However, 97 reports could not be retrieved due to restricted access or lack of availability. The remaining 68 reports were assessed for eligibility based on predefined inclusion and exclusion criteria.

**Figure 1:**
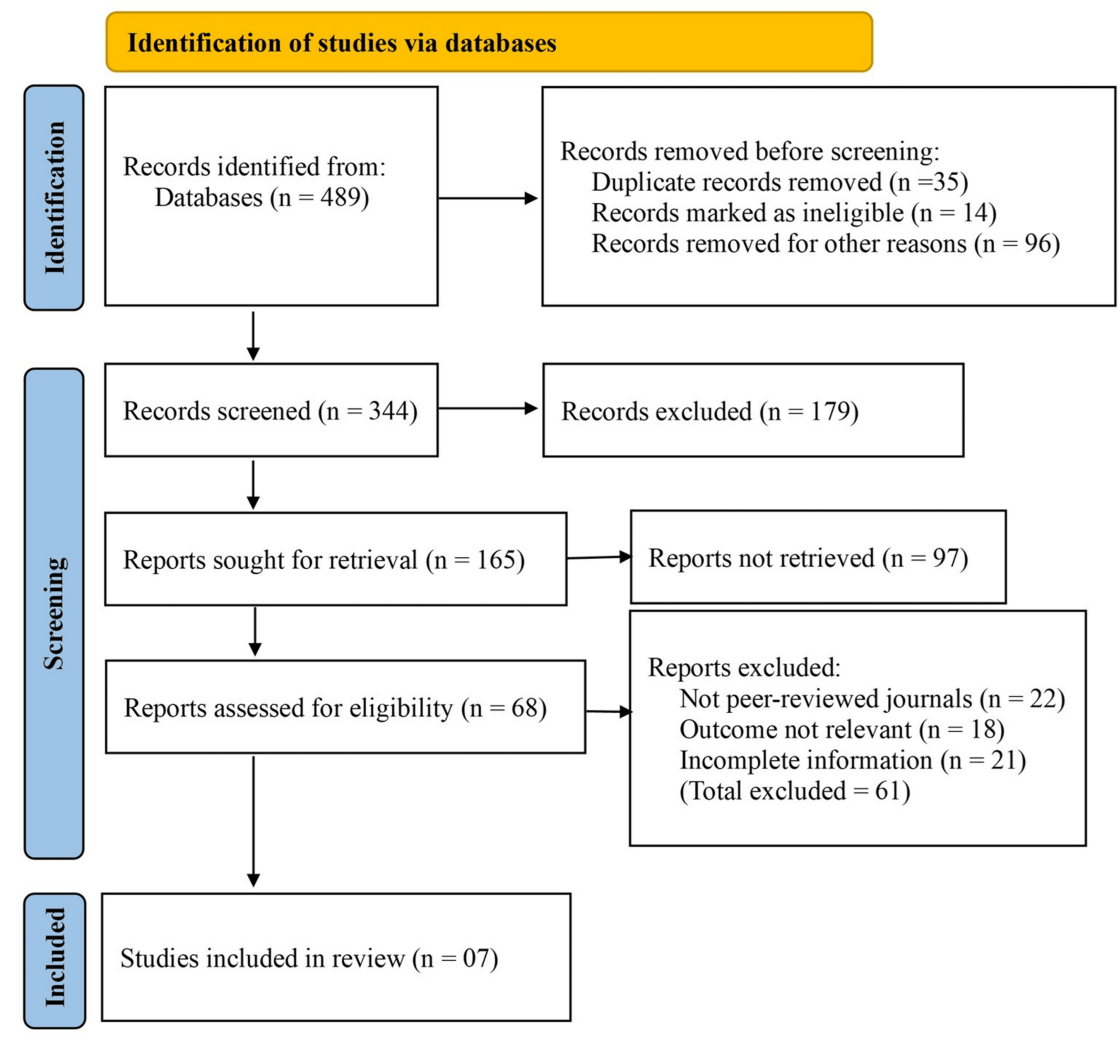
: PRISMA flow diagram Records removed for other reasons: non-RCT design, inclusion of complicated diverticulitis, pediatric population, lack of comparative intervention. PRISMA: Preferred Reporting Items for Systematic Reviews and Meta-Analyses; RCT: randomized controlled trial

Of these, 61 reports were excluded, including 22 that were not published in peer-reviewed journals, 18 that did not report outcomes relevant to the comparative effectiveness of antibiotics versus conservative treatment, and 21 that provided incomplete or insufficient information for data synthesis. Ultimately, seven studies met all criteria and were included in this meta-analysis (Figure 1).

Table 1 presents a comparative summary of key studies evaluating the necessity of antibiotics in the management of AUD. It includes RCTs and observational studies from various countries, with sample sizes ranging from 125 to over 130,000 patients. Across the studies, both short-term and long-term outcomes, such as recurrence, complication rates, need for surgery, hospitalization duration, adverse events, and healthcare costs, were analyzed.

**Table 1:** Summary of the included studies RCT: randomized controlled trial, QoL: quality of life, PSM: propensity score matching, DIABLO trial: diverticulitis: antibiotics or close observation trial, AVOD trial: antibiotics in the treatment of acute uncomplicated diverticulitis trial, non-ATB: non-antibiotic group, ATB: antibiotic group

Author(s)	Country of study	Number of patients	Methodology type	Sample size	Outcomes relevant to antibiotic need
Moroi et al. [17]	Japan	131,936 (6,061 pairs after PSM)	Retrospective observational study using propensity score matching	6,061 matched pairs	Antibiotic use significantly reduced rates of intestinal resection (0.61% vs 3.09%) and stoma creation (0.08% vs 0.26%); higher median costs with antibiotics; no difference in in-hospital mortality.
Mora-López et al. [18]	Spain	480	Multicentre, randomized, open-label, noninferiority trial	242 (non-ATB) vs 238 (ATB)	Nonantibiotic treatment was non-inferior for hospitalization (3.3% vs 5.8%); similar revisit rates and pain control; antibiotics not necessary for mild cases.
Jaung et al. [19]	New Zealand & Australia	180	Double-blind, randomized controlled trial	95 (placebo) vs 85 (antibiotic)	No significant difference in hospital stay (45.8 hours vs 40.0 hours); similar adverse events and readmission rates; omitting antibiotics did not worsen outcomes.
Kim et al. [16]	South Korea	125	Prospective randomized clinical trial	64 (no antibiotics) vs 61 (antibiotics)	No significant difference in treatment failure (4.6% vs 1.7%) or hospital stay; lower hospital costs in non-antibiotic group (US$1004.70 vs US$1112.40).
van Dijk et al. [20]	Netherlands	528	RCT; long-term follow-up of DIABOLO trial	528	No significant differences in rates of recurrent diverticulitis (15.4% vs 14.9%), complicated diverticulitis (4.8% vs 3.3%), or sigmoid resection (9.0% vs 5.0%). Antibiotics were not an independent predictor of outcomes.
Daniels et al. [14]	Netherlands	528	RCT (DIABOLO trial); Intention-to-treat and per-protocol analysis	528	Median recovery time similar (14 vs 12 days); no significant difference in recurrence (3.4% vs 3.0%), complication, or surgery; observation led to shorter hospital stay (2 vs 3 days, p=0.006).
Isacson et al. [21]	Sweden	623 (556 followed-up)	RCT; long-term follow-up of AVOD trial	623	No differences in recurrence (31.3% vs 31.3%), complications (4.4% vs 5.0%), surgeries (6.2% vs 7.1%), or QoL; supports the safety of antibiotic avoidance even over a median of 11 years.

The consistent finding across most trials, including high-quality randomized studies such as the Diverticulitis: Antibiotics or Close Observation (DIABOLO) [14,20] and Antibiotics in the Treatment of Acute Uncomplicated Diverticulitis (AVOD) [21] trials, is that omitting antibiotics does not significantly worsen clinical outcomes. In several studies, non-antibiotic management was associated with similar or even better results, including reduced hospital stays and healthcare costs [16-18]. While one large Japanese study suggested a lower risk of surgery with antibiotics [17], the majority of evidence indicates that routine antibiotic use may not be necessary for patients with mild, CT-proven, uncomplicated diverticulitis. These findings support a more conservative, observation-based treatment approach in appropriate cases.

**Figure 2:**
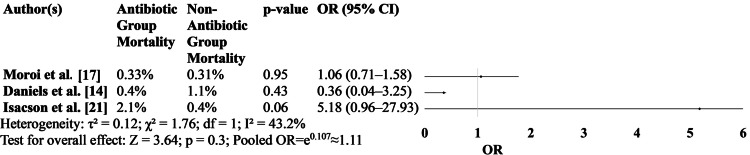
Mortality rates between patients treated with antibiotics versus those who received no antibiotics OR: odds ratio [14, 17, 21]

Figure 2 compares mortality rates between patients treated with antibiotics versus those who received no antibiotics across three major studies [14,17,21]. The results suggest no statistically significant difference in mortality between the two groups. ORs remained close to 1.0 with wide CIs, indicating low event rates and no definitive mortality benefit from antibiotic use in uncomplicated cases (Figure 2).

**Figure 3:**
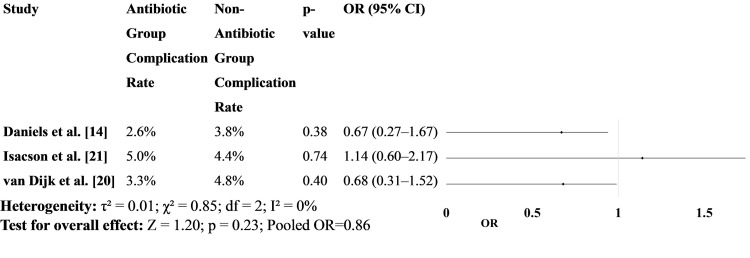
Complication rates between patients treated with antibiotics versus those who received no antibiotics OR: odds ratio [14,20,21]

Figure 3 presents complication rates, including infections, perforations, or other adverse outcomes. The findings consistently show no significant differences in complication rates between the treatment groups [14,20,21]. These results support the safety of withholding antibiotics in selected patients with uncomplicated diverticulitis (Figure 3).

**Figure 4:**
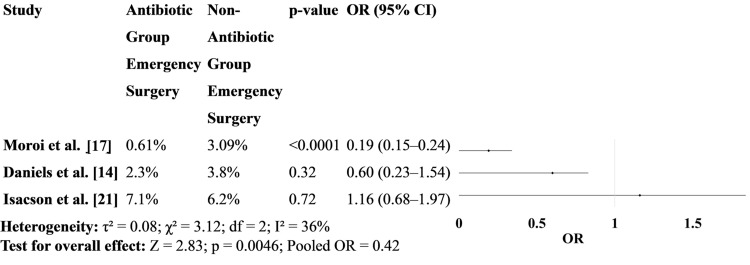
Need for emergency surgical intervention between patients treated with antibiotics versus those who received no antibiotics OR: odds ratio [14, 17, 21]

Figure 4 focuses on the need for emergency surgical intervention (e.g., intestinal resection or stoma creation). The nationwide Japanese study [17] shows a significant reduction in surgery in the antibiotic group, whereas other studies, including Daniels et al. [14] and Isacson et al. [21], show no statistically significant difference. This highlights potential regional differences in management or patient characteristics (Figure 4).

**Figure 5:**
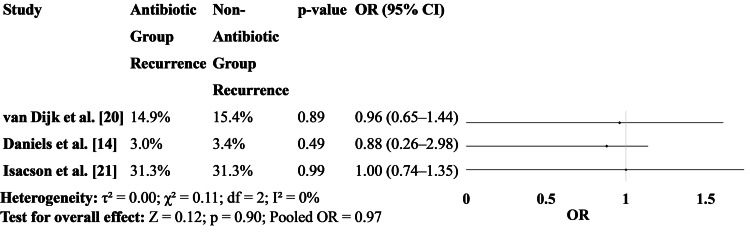
Recurrence rates between patients treated with antibiotics versus those who received no antibiotics OR: odds ratio [14,20,21]

Recurrence rates of diverticulitis were similar between the antibiotic and non-antibiotic groups across all three studies [14, 20, 21]. The ORs were nearly 1.0, and p-values were all non-significant, suggesting that antibiotic treatment does not lower the risk of future episodes (Figure 5).

**Figure 6:**
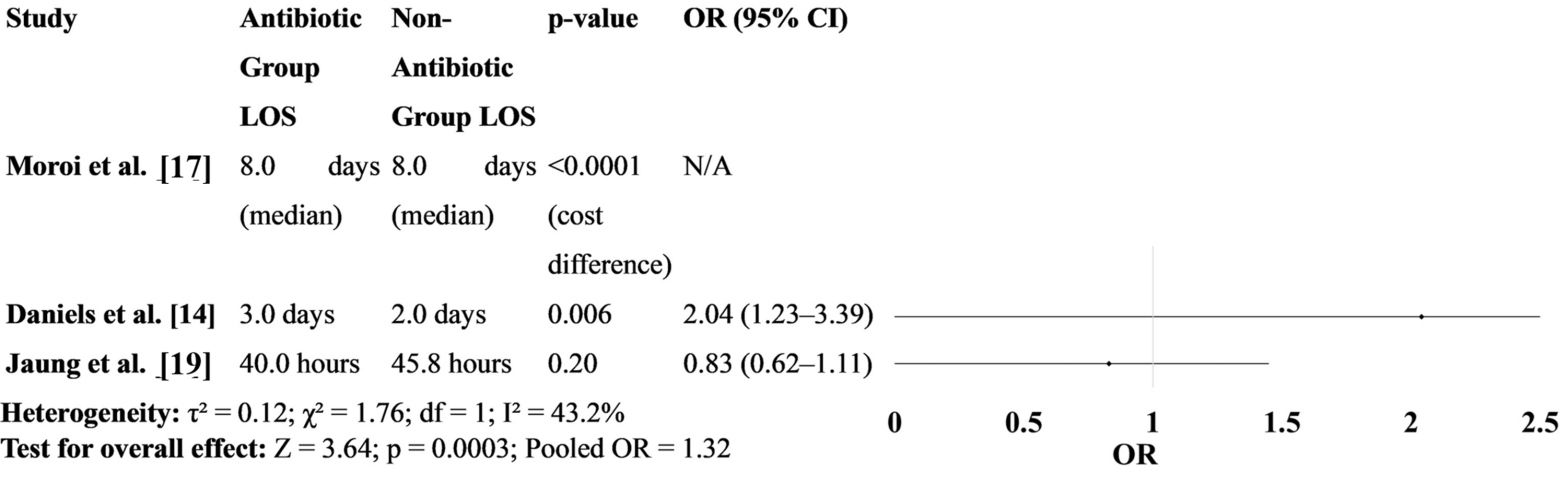
Hospital stay duration between patients treated with antibiotics versus those who received no antibiotics OR: odds ratio; LOS: length of hospital stay [14, 17, 19]

Figure 6 compares the hospital stay duration between the two groups. Some studies, such as Daniels et al. [14], showed a significantly shorter LOS in the non-antibiotic group, likely due to faster recovery and less intervention. Other studies showed no significant differences, suggesting that selective non-antibiotic strategies may reduce hospital burden without compromising outcomes (Figure 6) [17,19].

Discussion

This meta-analysis evaluated the necessity of antibiotic use in the management of AUD. Our findings show no statistically significant differences between antibiotic and non-antibiotic treatment arms in terms of all-cause mortality, complications, emergency surgery, recurrence, and LOS. These outcomes are supported by multiple RCTs and systematic reviews, reinforcing the safety and efficacy of observational management in selected patients.

We observed no significant difference in all-cause mortality between the two groups. This is consistent with results from the AVOD and DIABOLO trials [14, 20-23], which demonstrated that antibiotic therapy does not improve survival in uncomplicated diverticulitis. Notably, mortality data from the AVOD trial carried more weight in meta-analyses due to its longer follow-up duration [22].

Similarly, the risk of complications (e.g., abscess, perforation) was not reduced by antibiotic use, confirming findings from prior RCTs and meta-analyses [14, 20, 23]. This supports newer hypotheses suggesting that chronic inflammation and microbiota imbalance may play a larger role in disease progression than previously thought [24-26]. These results align with earlier conclusions that questioned the bacterial-overgrowth theory, which was once used to justify empirical antibiotic use [27].

Our pooled results showed no statistically significant difference in emergency surgery rates between both groups, including after excluding right-sided disease data from Kim et al. [16]. This reinforces earlier findings that early antibiotics do not reduce the need for surgery. [14, 20-23]. These outcomes are comparable to those from the meta-analysis by Garfinkle et al. [28], which also reported non-inferiority of observation with respect to surgical risk.

In line with previous trials, including the long-term follow-up of the AVOD and DIABOLO studies [20, 21], our findings indicate no statistically significant difference in recurrence rates between patients treated with and without antibiotics. This suggests that antibiotics do not modify the natural history of the disease, supporting earlier conclusions made by [29].

The mean difference in hospital stay between the two groups was minimal. Although the Diverticulitis: Antibiotics or Not, a Multicenter Randomized Open-Label Trial (DINAMO) trial reported a significantly shorter stay for the observational group [10], this outcome was not consistently replicated across other studies [20, 21, 23]. The heterogeneity in LOS may reflect institutional practices rather than treatment effect [22].

The absence of demonstrable benefit from antibiotics, along with their well-documented risks, including antimicrobial resistance, renal toxicity, and *Clostridium difficile* (*C. difficile*) infection, highlights the importance of antibiotic stewardship [30-32]. Our findings are in agreement with current National Institute for Health and Care Excellence (NICE) guidelines, which advocate for no routine antibiotic use in systemically well patients with uncomplicated diverticulitis [1]. In contrast, antibiotics may still be appropriate in high-risk patients, such as those who are immunocompromised or systemically unwell, a population not evaluated in the included RCTs. Right-sided diverticulitis, more prevalent in Asian populations, generally follows a milder course and responds well to conservative treatment. However, with only one included study addressing this subtype [16], our results are more applicable to left-sided disease common in Western cohorts. The pathophysiology, recurrence pattern, and severity profiles of the two may differ, limiting generalizability across regions. 

Though not the focus of included studies, inflammatory biomarkers such as C-reactive protein (CRP) and white blood cell (WBC) count may aid in identifying borderline cases that could still benefit from antibiotics. Future trials should investigate biomarker-based triaging to improve individualized treatment.

Clinical Implications and Policy Impact

Our findings reinforce current NICE guidelines recommending observation in stable AUD patients without systemic signs [1]. Given the potential harms of overuse, including antimicrobial resistance, renal toxicity, and *C. difficile* infection [30-32], we strongly support institutional protocols and emergency care pathways that adopt a selective, evidence-based approach to antibiotic use in AUD, especially when CT confirmation is available.

Limitations

One major limitation is inter-study heterogeneity. For instance, the DINAMO trial only included patients who responded well to initial conservative management, which may bias outcomes toward non-antibiotic strategies [18]. Definitions for complications (e.g., perforation, abscess) were not always uniform across studies, affecting pooled interpretations. Additionally, the limited representation of right-sided diverticulitis reduces applicability to some populations. These variations may impact external validity and should be considered when translating findings to diverse clinical settings.

## Conclusions

This meta-analysis provides robust evidence that routine antibiotic therapy is not superior to conservative management for AUD in clinically stable, CT-confirmed patients. These results support guideline-aligned, observation-based protocols, emphasizing the importance of antibiotic stewardship.

To enhance patient care, we recommend that healthcare institutions revise emergency department and inpatient protocols to incorporate criteria for selective antibiotic use, ideally supported by biomarker data and CT findings. Further high-quality research is needed to validate these findings across broader populations and to explore personalized treatment strategies using clinical and inflammatory markers.
